# A Young Lady With Myopericarditis: An Unusual Presentation of COVID-19 Infection

**DOI:** 10.7759/cureus.26673

**Published:** 2022-07-09

**Authors:** Md Nazmul Hasan, Adrita Afzal, Chowdhury Adnan Sami, Fazle R Chowdhury, Din-E-Mujahid M Faruque

**Affiliations:** 1 Internal Medicine, Bangabandhu Sheikh Mujib Medical University, Dhaka, BGD; 2 Cardiology/Interventional Cardiology, National Institute of Cardiovascular Diseases, Dhaka, BGD; 3 Cardiology, Bangabandhu Sheikh Mujib Medical University, Dhaka, BGD

**Keywords:** successfully treated myopericarditis, sars-cov-2, colchicine in myopericarditis, covid-19, myopericarditis

## Abstract

Fever, sore throat, cough, and shortness of breath are the characteristic clinical manifestations of coronavirus disease 2019 (COVID-19). As the epidemic spreads, it is evident that the infection can affect not only the lungs but also other organs. By attaching to the angiotensin-converting enzyme-2 receptor (ACE-2), the severe acute respiratory syndrome coronavirus 2 (SARS-CoV-2) induces lung injury. SARS-CoV-2 can also cause damage to the heart and blood vessels as these organs have abundant ACE-2 receptors. Here, we present a 28-year-old lady with shortness of breath, chest pain, low blood pressure, and a pulse rate that fluctuates widely. She had SARS-CoV-2-induced myopericarditis after further testing. Initially, we treated her with high-dose prednisolone and other supportive medications. Then, we also added colchicine and ibuprofen due to the initial poor response, and the result was satisfying after two weeks of treatment.

## Introduction

Since its emergence in Wuhan, China, coronavirus disease 2019 (COVID-19) has infected more than 8.6 million people, with over 13.5 thousand deaths in Bangladesh [1]. The typical manifestations of COVID-19 are fever, fatigue, and pulmonary symptoms [2]. However, various cardiac involvements, including myocardial infarction, myopericarditis, and arrhythmias, have been reported in the medical literature [3]. Zeng et al. described the first case of fulminant myocarditis and myocardial damage [3-4]. Myopericarditis is a rare and devastating complication of COVID-19 that can be easily missed. We present this case to emphasize the importance of vigilance so that physicians do not overlook myopericarditis in a COVID-19 patient.

## Case presentation

A 28-year-old woman presented to the COVID-19 dedicated medical ward with six days of fever, runny nose, exertional shortness of breath, palpitations, chest pain, and extreme weakness. She had no known co-morbidities. A reverse transcription-polymerase chain reaction (RT-PCR) for COVID-19 was positive. During admission, physical examination findings showed that her temperature was 100˚F, her blood pressure was 90/60 mm Hg, her pulse rate ranged from 50 to 140 beats per minute, and her respiratory rate was 30 breaths per minute. Although, her oxygen saturation in room air was 95% and remained normal. The only significant finding of the systemic examination was mild bilateral basal crepitation. 

**Figure 1:**
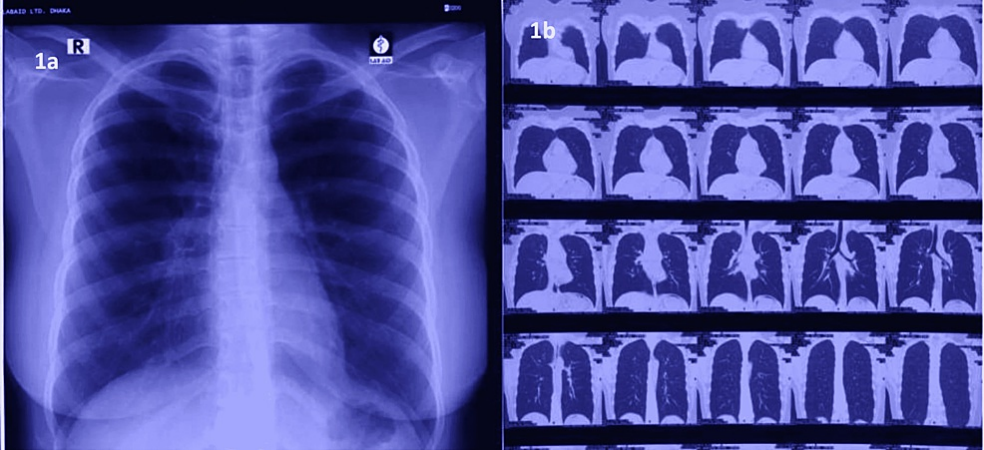
X-ray (a) and high-resolution computed tomography (HRCT) scan of the chest (b)

**Figure 2:**
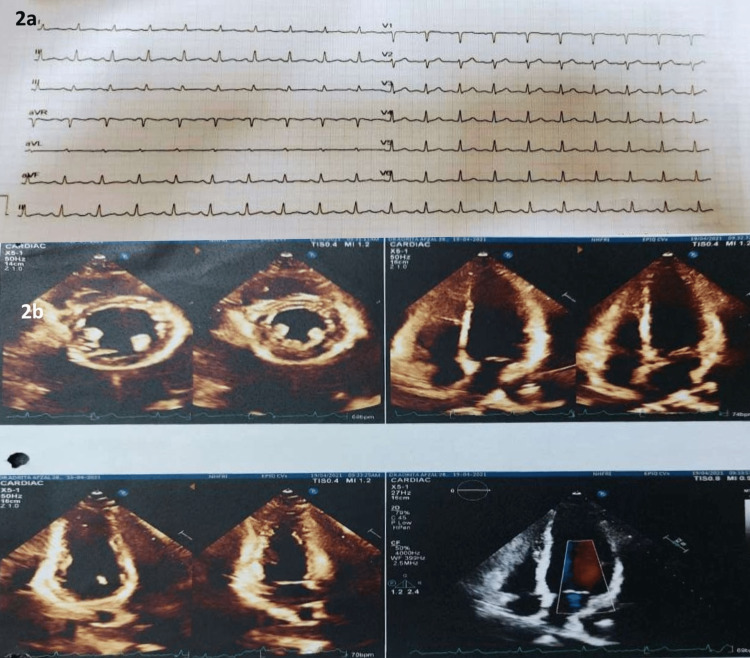
ECG demonstrated sinus tachycardia (a), echocardiography revealed normal wall motion and left ventricular ejection fraction (LVEF) of 60% (b)

**Figure 3:**
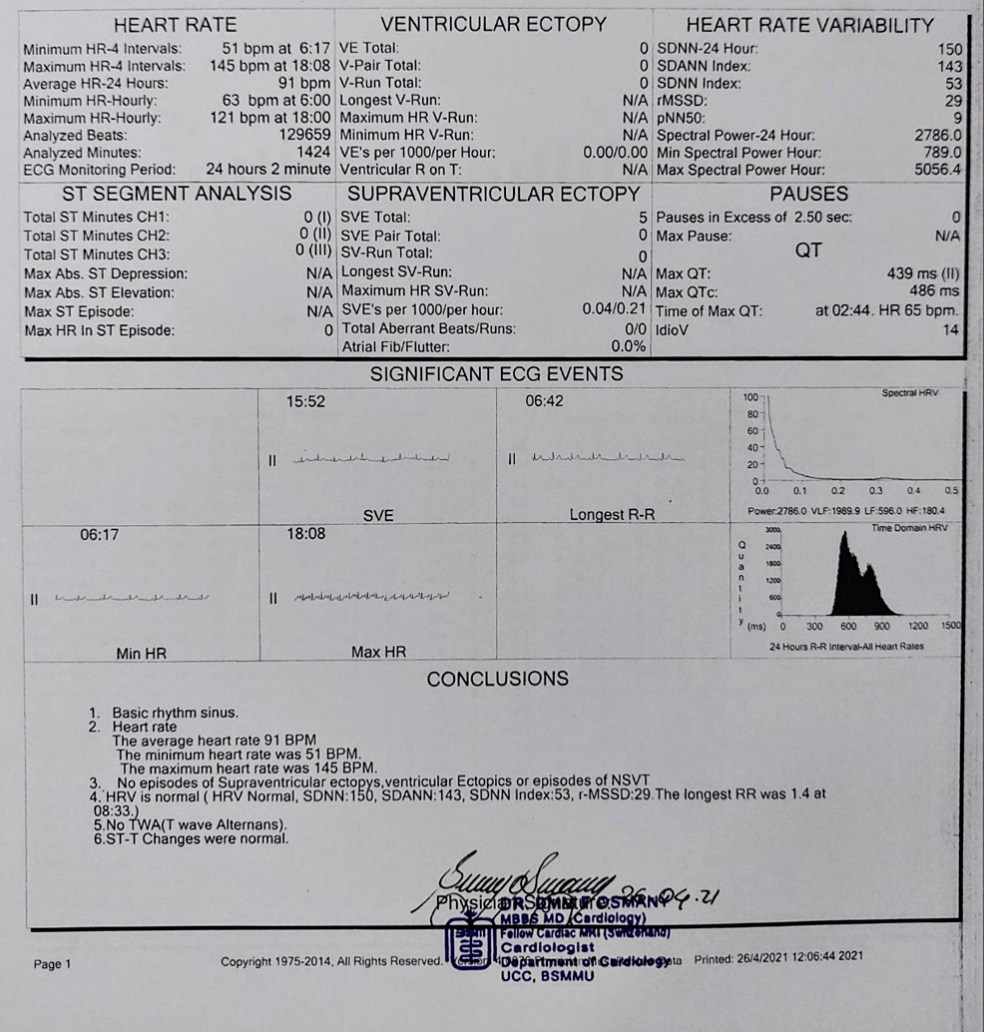
24-hour Holter monitoring revealed a sinus rhythm with an average rate of 91 beats per minute; the maximum heart rate was 145 beats per minute

**Figure 4:**
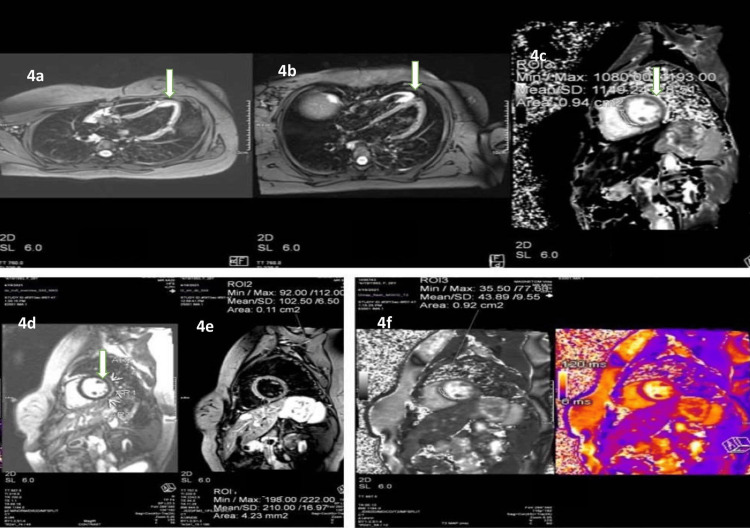
Cardiac magnetic resonance imaging showed hyperintensity signal changes in pericardium and myocardium

Her biochemical tests revealed lymphopenia and a high C-reactive protein (CRP). Nevertheless, her other inflammatory indicators, such as serum ferritin, D-dimer, lactate dehydrogenase (LDH), serum troponin-I, and brain natriuretic factor (pro-BNP), were normal (Table 1). The chest X-ray and high-resolution computed tomography (HRCT) scans were normal (Figures 1a, 1b). The electrocardiogram (ECG) demonstrated sinus tachycardia and a regular rhythm (Figure 2a). The echocardiography revealed normal wall motion at rest, a left ventricular ejection fraction (LVEF) of 60%, and an average left ventricular global peak longitudinal strain of 19.5% (Figure 2b). As a result of the fluctuating heart rate, a 24-hour Holter monitoring was performed, which revealed a sinus rhythm with an average rate of 91 beats per minute; the maximum heart rate was 145 beats per minute, and the minimum heart rate was 51 beats per minute (Figure 3). The cardiac magnetic resonance imaging (CMRI) showed hyperintensity signal changes in pericardium and myocardium in T-2 weighted and short tau inversion recovery (STIR) images, increased brightness (LGE) in a sub-epi-myocardial layer in phase-sensitive inversion recovery (PSIR), SI ratio over skeletal muscle 2.0, and prolonged global T-1 mapping time of 1149 ms (cut-off 1120 ms) and prolonged global T (Figure 4a-4f).

**Table 1:** Important biochemical and hematological parameters of the patient

Tests	Results (normal)
Hemoglobin	12.9 gm/dl
White blood cell count	5.83 X10^9^/L
Lymphocyte count (%)	14%
Neutrophil count (%)	78%
Platelet count	273 X10^9^/L
Creatinine	1.13 mg/dl
Alanine aminotransferase (ALT)	22 IU/l
Prothrombin time	14 seconds
C-reactive protein (CRP)	4.7 mg/dl (3 mg/dl)
Ferritin	18.6 ng/ml (10-291 ng/ml)
Lactate dehydrogenase (LDH)	240 U/L
D-dimer	0.44 mcg/ml (<0.50 mcg/ml)
Troponin-I	<0.01 ng/ml (0.017-0.056 ng/ml)
Creatinine kinase (CK-MB)	24 IU/L (3-25 IU/L)
Pro-brain natriuretic factor (pro-BNP)	35.5 pg/ml (up to 178 pg/ml)

Therefore, myopericarditis caused by COVID-19 was identified. She was treated with corticosteroids and beta-blockers and observed for one week with no discernible improvement. In addition to steroid treatment, she received ibuprofen 400 mg thrice daily and colchicine 0.6 mg twice daily for 14 days. She responded satisfactorily to treatment and was stable over the follow-up period.

## Discussion

The clinical presentation of COVID-19 varies from mild illness to major system involvement with fatal outcomes. In addition to other system involvement, the SARS-CoV-2 infection has been linked to cardiovascular complications such as myocardial infarction, thromboembolic events, and myopericarditis [3]. Sala et al. reported the first case of biopsy proved myocarditis in a COVID-19 patient [5]. There are two possible mechanisms to cause myocardial damage. Firstly, the SARS-CoV-2 virus directly inoculates into the myocardium by binding to transmembrane serine receptor-2 (TMPRSS2) and causes inflammation. Secondly, T lymphocyte-mediated cellular immune response to SARS-CoV-2 viral infection causes inflammation by cell-mediated cytotoxicity [6].

Many studies have suggested that myocarditis can present without typical respiratory symptoms, as in our case [7]. The clinical features of myopericarditis may be similar in COVID-19 as in other patients. In a review, Kariyanna et al. described dyspnea as the predominant clinical feature, followed by coughing, fever, and chest pain as the presenting symptoms in COVID-19-associated myopericarditis [8]. Another study found that 64% of patients with myocarditis in COVID-19 had presented with shock [9]. Our patient presented with fever, chest pain, shortness of breath, low blood pressure, and fluctuating heart rate, which is consistent with the literature.

For the diagnosis of myopericarditis in COVID-19, other possibilities need to be ruled out by history and investigations. The temporal relation between the symptoms and RT-PCR positivity proved this case. The coronary angiogram may be required in some cases to exclude other causes. An endo-myocardial biopsy is the gold standard for isolating viral particles from the myocardium [10-11]. The studies related to COVID-19 myopericarditis showed variable electrocardiographic (ECG) findings, a rise of troponin-I in about 91% of patients, and the LVEF was reduced in 60% of the patients [9]. Kariyanna et al. reported that pro-BNP was elevated in all analyzed studies [8]. In this case, we found normal troponin-I, pro-BNP, and ECG revealed sinus tachycardia and LVEF was normal in echocardiogram without wall motion abnormalities. Ultimately, CMRI confirmed the diagnosis of myopericarditis. Unfortunately, we could not do an endomyocardial biopsy due to the test’s unavailability, which is probably a limitation.

The treatment of viral myocarditis is supportive. Some authors advised using a high dose of steroids and immunoglobulin intravenously (IVIG) in COVID-19-associated myocarditis [12]. However, antiviral medication has an unproven role in treating myocarditis [13]. Individual cases were managed with colchicine 500 µgm twice daily and ibuprofen 400 mg thrice daily with prednisolone 30 mg daily with good results [14]. We also treated her with colchicine 600 µgm twice daily, ibuprofen 400 mg thrice daily, and steroid. She had significant improvement in her symptoms after two weeks of the treatment.

## Conclusions

The outcome of myopericarditis in COVID-19 patients may be fatal and can cause rapid deterioration of the clinical condition. In our case, early identification, prompt treatment, and careful patient monitoring led to a satisfying outcome for the patient. So, the physician must be stringent about identifying the cases of myopericarditis as early as possible to salvage the life of the patients.
